# Digestomics of Cow’s Milk: Short Digestion-Resistant Peptides of Casein Form Functional Complexes by Aggregation

**DOI:** 10.3390/foods9111576

**Published:** 2020-10-30

**Authors:** Jelena Radosavljević, Danijela Apostolović, Jelena Mihailović, Marina Atanasković-Marković, Lidija Burazer, Marianne van Hage, Tanja Ćirković Veličković

**Affiliations:** 1 Center of Excellence for Molecular Food Sciences & Department of Biochemistry, Faculty of Chemistry, University of Belgrade, 11000 Belgrade, Serbia; radosavljevic@chem.bg.ac.rs (J.R.); vesic@chem.bg.ac.rs (J.M.); 2 Immunology and Allergy Unit, Department of Medicine Solna, Karolinska Institute and University Hospital, 17164 Stockholm, Sweden; danijela.apostolovic@ki.se (D.A.); marianne.van.hage@ki.se (M.v.H.); 3 Department of Allergology and Pulmonology, University Children’s Hospital, Belgrade, Serbia; marinaamarkovic@gmail.com; 4 School of Medicine, University of Belgrade, 11000 Belgrade, Serbia; 5 Torlak Institute of Immunology and Virology, Vaccine and Sera Production, 11152 Belgrade, Serbia; slitor321@yahoo.com; 6 Department of Environmental Technology, Food Technology and Molecular Biotechnology, Ghent University Global Campus, Yeonsu-gu, Incheon 21985, Korea; 7 Faculty of Bioscience Engineering, Ghent University, 9000 Ghent, Belgium; 8 Serbian Academy of Sciences and Arts, 11000 Belgrade, Serbia

**Keywords:** milk, allergenicity, allergy, pepsin, casein, IgE, digestion-resistant peptides, gastric simulated digestion

## Abstract

The aim of this study was to identify short digestion-resistant peptides (SDRPs) released by pepsin digestion of the whole cow’s milk and examine their IgE reactivity and allergenicity. Raw milk was subjected to simulated gastric digestion. SDRPs were fractionated from the digests and identified by MS. Milk SDRPs were evaluated for aggregability, propensity to compete for IgE binding with individual milk allergens, and ability to bind IgG4 from allergic and milk-tolerant individuals. The majority of milk SDRPs originated from caseins (97% of peptides) and overlapped with the known IgE epitopes of cow’s milk allergens. SDRPs competed with milk proteins for binding to human IgE and readily formed aggregates. The average peptide length was 10.6 ± 3.5 amino acids. The ability to provoke allergenic in vivo responses was confirmed by skin-prick testing (SPT) in five milk-allergic subjects. This was attributed to the peptide ability to aggregate into non-covalent complexes. SDRPs are able to induce response in SPT, but only in 50% of the sera SDRPs were able to inhibit IgG4 binding to caseins. Hence, SDRPs corresponding to the mainly continuous epitopes of milk proteins induce allergenic in vivo responses in milk-allergic subjects due to aggregation.

## 1. Introduction

Cow’s milk is a major cause of food allergy in infancy with approximately 2.5% of young children manifesting this type of allergy [1,2]. The majority of the affected children (79%) outgrow milk allergy by the age of 16 years, leaving only 1% of adult population suffering from this condition [3]. The only method of management of cow’s milk allergy (CMA) symptoms is strict avoidance of dairy products. For young children, hypoallergenic milk formulae based on extensively hydrolyzed milk proteins are proposed as alternative forms of milk that do not cause adverse reactions. The criteria introduced by European Academy for Allergy and Clinical Immunology for labelling the milk formulas as hypoallergenic relies on the clinical tolerance of proposed formula in 90% of the milk-allergic children (with 95% confidence interval) [4]. However, currently there is no agreement on the molecular weight of the peptides in milk formula that can be used as a criterion for labeling formula as hypoallergenic. Generally, the molecular weight of 1500 Da is included in regulations as an upper limit of molecular weight of peptides in hypoallergenic formulas that is considered as a safe for use in nutrition of children with CMA [4,5].

Resistance to gastrointestinal digestion is an important factor of protein allergenicity. Many procedures for the assessment of allergenicity measure the sensitivity of food proteins to gastric (or gastrointestinal) digestion in order to predict their allergenic properties have been proposed [6,7,8,9]. Although earlier reports implied that the resistance to pepsin digestion is crucial for allergenicity, further studies revealed that there was no clear relationship between those two properties [10,11,12].

At present, a positive correlation between allergenicity and proteolytic stability is implied by the regulations for the assessment of newly introduced proteins in genetically modified foods [8,13]. However, recent studies that used human digestive juices and animal digestion models questioned the validity of the previously adopted procedures [14,15,16]. The standardized in vitro gastrointestinal digestion protocol provides conditions considered as physiologically relevant [17]. The complexity of the matrix in which proteins encounter digestive proteases also affects protein digestion stability. Lipids and polysaccharides present in the digestion mixture usually attenuate protein degradation in digestion tests [11,18,19]. For example, the major peanut allergens Ara h 1 and Ara h 3 were estimated to be 500-fold less digestible by pepsin in the whole peanut grain than in solution [19]. These studies provided an additional proof that allergenicity of labile proteins determined in simple digestion tests on purified proteins cannot be solely attributed to the cross-reactivity of digestion-resistant allergens.

The initial simplicity in the assessment of the food allergens’ resistance to proteolysis has evolved into the complex pipeline for the evaluation of the proteins’ digestion fate in foods, termed digestomics. This approach relies on use of sophisticated methodologies such as high resolution mass spectrometry for identification of the peptides surviving proteolysis, as well as one- and two-dimensional electrophoresis combined with mass spectrometry for monitoring the digestion fate of the proteins in complex food mixtures and proteolysis survival of larger protein fragments, which are known to exhibit allergenic properties [20].

Besides proteolytic stability, the size of digestion-resistant peptides and their physicochemical properties are likely crucial for allergenicity. Initially, it has been postulated that peptides <3 kDa are ignored by the immune system as they cannot sensitize or induce allergic reaction in previously sensitized animals [21]. However, later studies demonstrated that allergenic properties are preserved in digestion-resulting peptides [22], especially if they are held together and able to adopt three-dimensional structures similar to that of the native allergen [12,19,23].

Many studies investigated digestion of milk proteins under different experimental conditions. Frequently, purified proteins [14] or milk protein preparations were used [16]. Here, we have conducted, to the best of our knowledge, the first digestomic study addressing pepsin digestion of allergens in raw milk, i.e., in the lipid- and protein-rich matrix. Our goal was to identify those intact proteins and peptides that survive gastric digestion and to examine IgE and IgG4 reactivity of short digestion-resistant peptides (SDRPs) of milk.

## 2. Materials and Methods

All chemicals, if not otherwise stated, were purchased from Sigma-Aldrich (Hamburg, Germany). Raw, thermally untreated milk was obtained from a local farm.

### 2.1. Digestion of Raw Milk in Simulated Conditions of the Stomach

Digestion of raw milk was done at 37 °C as follows: pH of a milk aliquot was adjusted to 3.6 by 3 M HCl, and pepsin was added to obtain activity of 2375 U/mL. The reaction was stopped by adding 2 M sodium carbonate at different time points up to 4 h. Samples were defatted twice by tetrachloroethylene extraction (sample:tetrachloroethylene = 3:1 (v:v), followed by centrifugation for 10 min at 13,400 rpm. Defatted samples were kept frozen at −80 °C.

Digestion profiles were obtained by electrophoresis on 16% polyacrylamide gels. Control sample contained the same amount of 3 M HCl and pepsin as the digestion mixture, and pH was set to 3.6 by adding 6 M NaOH.

### 2.2. Detection of α-Lactalbumin (ALA) and β-Lactoglobulin (BLG) by Immunoblotting

Stopped digests were mixed with reducing sample buffer and boiled for 5 min at 95 °C. ALA and BLG were resolved on 16% polyacrylamide gel with molecular weight markers (Fermentas, Vilnius, Lithuania) and detected by rabbit anti-whey primary antibodies and anti-rabbit secondary antibody coupled to alkaline phosphatase (ABD Serotec, Oxford, UK). For details, see Supplementary Information.

### 2.3. Preparation of Milk SDRPs

Raw milk was digested for 1 h in simulated conditions of the stomach (as described in Section 2.1: at 37 °C, pH = 3.6, and pepsin activity of 2375 U/mL) and, after the reaction was stopped by sodium carbonate, the obtained digest was frozen at −20 °C. Digested milk was defatted as described above and sequentially filtered through Amicon centrifugal filtration units with decreasing cut-offs of 50, 30, 10, and 3 kDa, respectively (Merck Millipore, Darmstadt, Germany). Flow-through fraction obtained by the centrifugal filtration through a 3-kDa Amicon centrifugal filtration unit was considered as milk-derived SDRPs with the mass <3 kDa (MPs). Control sample contained only the peptides originating from pepsin (PP) autoproteolysis and was prepared similarly to milk-derived SDRPs. Peptides were lyophilized and stored at −20 °C until further use.

### 2.4. Mass Spectrometry Analysis

MPs were purified by Supel-Tips C18 Pipette Tips (Supelco, Sigma-Aldrich, Munich, Germany) according to the manufacturer’s instruction. Upon drying, the samples were dissolved in 0.1% formic acid and analyzed with Easy nanoLC II coupled to LTQ Orbitrap XL (Thermo Scientific, Bremen, Germany). For details, see Supplementary Information.

### 2.5. Patients

Sera from 11 Swedish milk-sensitized patients and one Serbian milk-sensitized patient with IgE levels to whole milk in the range of range 11–415 kUA/L (Supplementary Table S1, patients #1–#12) (ImmunoCAP System; Phadia/Thermo Fisher Scientific, Uppsala, Sweden) were used for IgE inhibition. A total of five Serbian milk-allergic patients were selected for skin-prick test (SPT), and their sera were used for the IgG4 inhibition study (#12–#16, Supplementary Table S1). Serum samples from five non-allergic individuals (<0.3 ISU-E to milk components by ISAC ImmunoCAP and <0.1 kUA/L to casein by ImmunoCAP) were used as controls in the IgG4 inhibition study (#17–#21, Supplementary Table S1). As controls for SPT, two Serbian patients (#22 and #23, Supplementary Table S1) with history of allergy to inhalatory weed allergens, but without history of milk allergy have been selected as controls, based on negative SPT with commercial mixture of milk allergens.

Experiments involving Serbian patients were approved by the Ethical Committee of the University Children’s Hospital, Belgrade, Serbia (number 017/6-990/6) and performed according to Serbian National guidelines (which follow the Declaration of Helsinki) for studies involving human subjects. Informed written consent was obtained prior to the study.

Experiments involving sera of Swedish individuals were selected at the Department of Clinical Immunology of the Karolinska University Hospital. All experiments were in accordance with relevant guidelines and regulations. Because of the retrospective nature of the study, no additional consent was required for IgE binding tests.

### 2.6. Hydrophobicity Calculations

Hydrophobicity plots were made by ProtScale web application available at ExPASy (www.expasy.org), using the hydrophobicity scale of Kyte and Doolittle [24], applying the window size of 11 amino acids and linear weight variation model without normalization of the scale.

### 2.7. Size-Exclusion Chromatography (SEC)

For the SEC details, see Supplementary Information.

### 2.8. Electrophoretic Analysis of SDRPs

Electrophoretic analysis of the peptides <3 kDa was done on Mini-PROTEAN Tris-Tricine and 4–20% Mini-PROTEAN TGX precast gels (Bio-Rad, Hercules, CA, USA).

### 2.9. IgE-Binding Properties of SDRPs

Lyophilized MPs and PPs were reconstituted in 5-fold lower volume of water. For assessing the capacity of digestion-derived peptides to inhibit binding of IgE to intact milk proteins, ALA, BLG, or casein fraction, peptide fractions were incubated overnight at 4 °C with sera of milk-allergic persons at the 1:1 (v:v) ratio. Prior to the incubation with MPs or PPs, some sera were diluted with ImmunoCAP IgE sample diluent (Phadia AB/Thermo Scientific) (final dilutions after mixing with peptides are given in Supplementary Table S1). Sera were incubated with peptides and levels of IgE antibodies to milk (f2), casein (78), ALA (f76), and BLG (f77) were measured by an ImmunoCAP 1000 System (Phadia AB/Thermo Scientific). Reactions to milk were analyzed in patients 1–12, whereas reactions to casein, ALA, and BLG were analyzed in patients 3–12. Maximal (non-inhibited) binding was measured in sera preincubated with PPs prepared similarly to MPs. Inhibition of IgE binding was expressed as percentage based on maximal binding, using the following formula: % IgE inhibition = 100 − ((IgE binding to the solid surface in the presence of MP/IgE binding to the solid surface in the presence of PP) × 100).

### 2.10. IgG4-Binding Properties of SDRPs

For IgG4-binding, sera of five milk-allergic (patients 12–16) and five milk-tolerant (patients 17–21) individuals were diluted 10-fold and preincubated with PPs or MPs as in IgE-binding experiments. To detect IgG4 binding to α-, β- or κ-casein-coated plates, a detection antibody from an IgG4 Human Uncoated ELISA Kit with Plates (Cat. No # 88-50590-22, Invitrogen, Life Technologies Corporation, Carlsbad, CA, USA) was used. For details, see Supplementary Information.

### 2.11. Skin-Prick Tests (SPTs)

For patients 12–16 and 22–23, SPTs with commercial mixture of milk allergens (Torlak Institute, Belgrade, Serbia), MPs, and PPs as well as with 10 mg/mL histamine as positive control and saline solution as negative control were performed. Wheal size was measured after 15 min and calculated as the average of the longest diameter and the diameter perpendicular to it.

### 2.12. Statistical Analysis

Statistical analysis (paired Student’s *t*-test) was done by GraphPad Prism software (La Jolla, CA, USA) with a significance level of α = 0.05 (*p* < 0.05).

## 3. Results

### 3.1. Raw Milk Pepsin Digestion Reveals Intact Proteins

**Figure 1:**
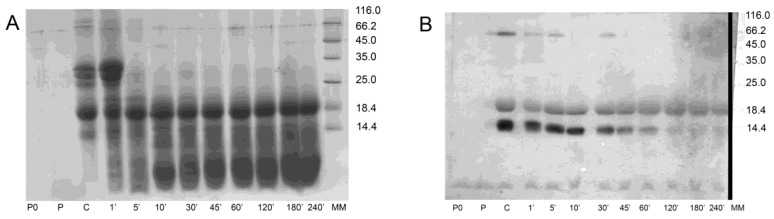
Pepsin digestion of raw milk over a 4-h period: (**A**)—SDS-PAGE, (**B**)—Western blot probed with antibodies against whey proteins. P0—pepsin digestion control stopped after 0 min; P—pepsin digestion control stopped after 240 min; ©—milk control sample (with no pepsin added); 1′, 5′, 10′, 30′, 45′, 60′, 120′, 180′, 240′—milk samples digested with pepsin for 1, 5, 10, 30, 45, 60, 120, 180 and 240 min, respectively; MM—molecular weight markers (indicated in kDa).

Digestion of raw milk proved that proteolysis of ALA was attenuated (Figure 1A) in comparison to that of the purified protein (Figure S2). Digestion of BLG was not affected by the food matrix (Figure S1), and that protein was resistant to the degradation during the 4-h period of raw milk digestion by pepsin (Figure 1). Degradation fragments of less resistant milk proteins accumulated over time and were visible in SDS-PAGE with a mass of <14 kDa. These degradation fragments did not exhibit reactivity to anti-whey antibodies (Figure 1B).

### 3.2. Peptide Analysis by High-Resolution Mass Spectrometry

**Figure 2:**
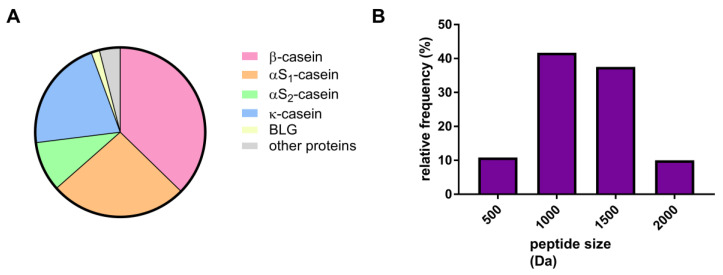
Analysis of the peptides identified after in vitro digestion of milk: (**A**)—protein origin distribution, (**B**)—peptide size distribution profile.

Peptides obtained by in vitro digestion were derived from β-casein (37.3%), αS1-casein (26.1%), αS2-casein (9.5%), and κ-casein (21.4%) (Figure 2A). BLG-derived peptides were present as a minor fraction (1.6%) (Figure 2A). ALA-derived peptides were not identified.

The majority of SDRPs were less than 2000 Da in size (1227.52343 ± 376.11328 Da (mean ± SD)). SDRPs ranging from 500 to 1500 Da accounted for 90.0% of all peptides identified after in vitro digestion (Figure 2B and Figure S3).

**Figure 3:**
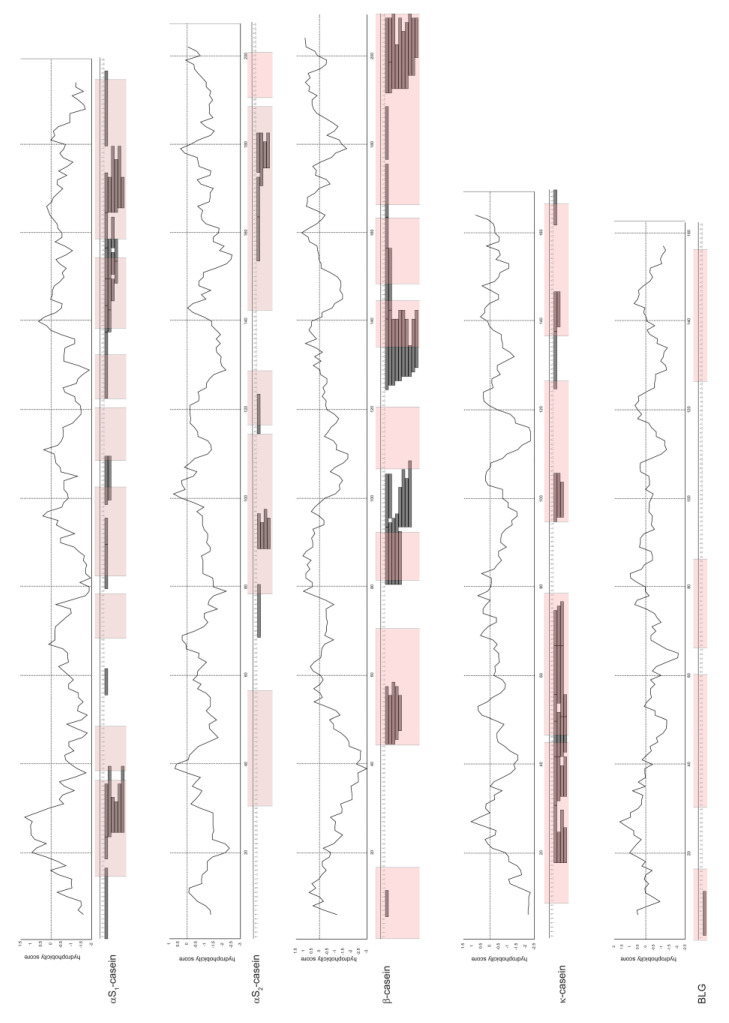
Overlapping of identified digestion-resistant peptides of cow’s milk proteins and hydrophobicity indexes of the proteins calculated by ProtScale web application available at ExPASy (www.expasy.org). Pink-shaded regions are known IgE-binding epitopes [25,26,27].

Alignment of identified peptides with sequences of major milk allergens revealed that majority of the peptides surviving digestion were derived from the same regions of milk proteins (Figure 3). Moreover, those regions frequently overlapped with previously reported IgE-binding milk protein epitopes [25,26,27,28]. Some identified peptides have previously been reported as surviving gastric digestion with simulated and human digestion fluids (Supplementary Table S2) [14]. Moreover, the peptides YQEPVLGPVR and VAPFPEV derived from β-casein (193–202) and αS1-casein (25–31) were previously identified among the peptides, which could be transported across Caco-2 cell monolayer [29].

### 3.3. SDRPs Originating from Milk Allergens Aggregate into Higher Molecular Weight Complexes

**Figure 4:**
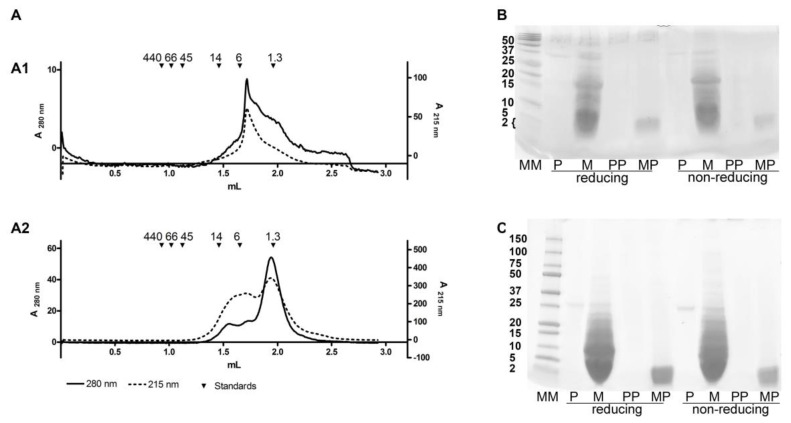
Detection of aggregation: (**A**)—size exclusion chromatography (SEC) profiles of PP (**A**1) and MP (**A**2), (**B**)—tricine electrophoresis, (**C**)—SDS-PAGE in 4–20% gels. P—pepsin autoproteolysis sample stopped after 60 min, M—milk digestion sample stopped after 60 min, MP—milk-derived peptides <3 kDa, PP—pepsin autoproteolysis peptides <3 kDa; MM—molecular weight markers (indicated in kDa).

SEC analysis of SDRPs with the mean size of 1.2 kDa revealed that they tended to form aggregates of larger sizes in physiologically relevant conditions. SEC profile showed that peptides formed complexes of >6000 Da (Figure 4A), suggesting that ≥4 peptides aggregated together and formed a large complex. Integration of the area under the curve at 215 nm showed that 43.5% of the peptides aggregated into larger complexes (Figure 4A). The electrophoretic profile of milk peptides was not altered by the presence of reducing agents (Figure 4B,C). Therefore, the aggregates are formed solely due to hydrophobic interactions between the peptides and/or attractive ionic interactions between positively and negatively charged functional groups of the peptides.

Peptide aggregability frequently correlates with the hydrophobicity of their amino acid residues. Therefore, we plotted the identified peptides of cow’s milk allergens versus the hydrophobicity index of their constituent amino acids (Figure 3). Our data showed that positive hydrophobicity index could be associated with the regions of β-casein that were enriched with SDRPs, and the N-terminal region of BLG (Figure 3).

### 3.4. SDRPs Associate into Functional Complexes, Bind IgE, and Do Not Contribute to the Tolerance to Milk Allergens

**Figure 5:**
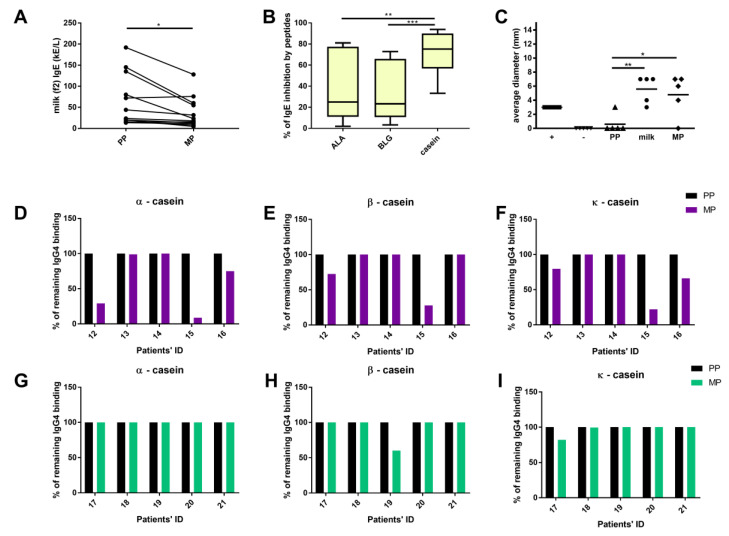
IgE-binding properties of milk-derived peptides: (**A**)—IgE binding to the milk protein-coated solid phase (f2) in the presence of pepsin peptides (PP) or milk-derived peptides (Ms), (**B**)—inhibition of IgE binding to ImmunoCAPs coated with individual milk proteins by MP, (**C**)—skin-prick testing (SPT): average wheal diameter upon the tests with: saline (−), histamine (+), pepsin peptides (PP), commercial milk protein extract (milk), and milk-derived peptides (MP), (**D**–**F**)—inhibition of IgG4 binding to caseins by MP in five milk-allergic patients tested in SPT: (**G**–**I**)—inhibition of IgG4 binding to caseins by MP in five milk-tolerant patients. * *p* < 0.05, ** *p* < 0.005, *** *p* < 0.001.

SDRP diminished human IgE binding to commercially available ImmunoCAPs coated with milk protein extract (Figure 5A). In addition, SDRPs inhibited binding of IgE to IimmunoCAPs coated with caseins more prominently than to ImmunoCAPs coated with ALA or BLG (Figure 5B). These findings correlated with digestomics data as the majority of identified peptides originated from caseins (94.3% of all SDRPs).

Furthermore, the ability of the obtained peptides to elicit allergic response was tested in SPT as a proof-of-concept. In four out of five tested patients, the reactions to the peptides were comparable to those in response to commercial milk extract (Figure 5C). The control subjects with no history of milk allergy did not reacted in SPT to commercial milk protein preparation, as well as to milk SDRPs.

IgG4 binding to SDRPs was analyzed by inhibition ELISA using sera from five individuals allergic to cow’s milk and five milk-tolerant individuals. In serum samples of three of the five individuals allergic to cow’s milk, SDRPs inhibited IgG4 binding to α-, β-, and/or κ-caseins (patients #12, #15, and #16; Table S1 and Figure 5D–F). Patient #15 that did not respond with a positive SPT to SDRPs demonstrated the highest inhibition of IgG4 binding to all three caseins tested among the tested patients. In half of the serum samples of SPT responders, SDRPs did not bind IgG4.In serum samples from five milk-tolerant individuals, the presence of SDRPs did not affect the binding of IgG4 to α-caseins (Figure 5G). Inhibition of the IgG4 binding to the β-caseins in the presence of MP was observed only in the serum from the one milk-tolerant individual (Patient #19, Figure 5H). The presence of MP affected the binding of IgG4 to κ-caseins in the serum samples of two milk-tolerant individuals (Patient #17 and slightly patient #18, Figure 5I). These findings prove that milk SDRPs do not contribute to the tolerance to caseins.

## 4. Discussion

One of the main approaches to diminish the ability of certain foods to provoke allergic reaction is extensive hydrolysis of the proteins to the extent at which they cannot induce immune response. However, a clear correlation between the degree of hydrolysis and the extent of allergenicity reduction has not been established yet. Our study confirmed that during pepsin digestion of raw milk BLG and ALA remained intact, whereas SDRPs originated from unstructured caseins and bound IgE. Most importantly, we showed that SDRPs possess functional allergenic properties due to their aggregation propensity.

Recently, in vitro digestion assays received much criticism mainly because their conditions did not reflect the conditions in vivo [6]. More importantly, because proteolytic stability was tested in purified proteins, in vitro assays usually neglected the effect of food matrix on proteins’ digestibility. Here, we assessed how components of raw milk were digested in simulated conditions. Our protocol was in compliance with the international consensus on physiologically relevant, static, in vitro digestion method [17].

We confirmed that BLG and ALA survived digestion in raw milk and apparently remained intact (Figure 1 and Figure S2). BLG is generally known as a pepsin-resistant protein, whereas purified ALA is easily degraded in vitro (Figures S1 and S2) [7,9]. Our data suggest that in a complex mixture such as raw milk, digestion of ALA is attenuated (Figure 1 and Figure S2). Moreno and co-workers have showed that the presence of phospholipid vesicles impaired digestion of ALA by pepsin in vitro [11]. ALA binds to different molecules: lipids, vitamins, natural phenolic and hydrophobic peptides [30,31,32]. Hence, we assume that ALA association with the lipid-rich matrix and, to some extent, with hydrophobic peptides released during digestion of caseins were the crucial factors that protected ALA from pepsin digestion in raw milk (Figure 3). We have also confirmed that pH change does not affect the susceptibility of purified ALA to digestion by pepsin (Figure S2).

The majority of the peptides detected in digests derived from caseins. Only few peptides derived from BLG were identified, and none from ALA, confirming the notion that those proteins mostly resisted digestion, but did not generate other digestion-resistant peptides. The proportion of the peptides detected in digests derived from individual casein fractions to some extent reflected the abundance of caseins in the milk: αS1-casein, 32%; αS2-casein, 10%; β-casein, 28%; κ-casein, 10% (Figure 2A) [33]. Our results were in line with published data on in vivo digestion of skim-milk powder. Notably, for the pepsin-susceptible αS1-casein, many peptides were present at the very beginning of the gastric phase, but their number decreased over time. In contrast to that progression, only a few peptides from β-lactoglobulin were generated during the whole gastric phase [34].

We compared the peptides from in vitro digestion with those that were shown to be transported across Caco-2 cell line monolayer, which is used as a model of the transport over the intestinal barrier of the food components [29]. Only few peptides reported in study of Picariello and co-workers as successfully transported [29] were found in in vitro gastric digests in our study (Table S1). These findings were expected, as Picariello and co-workers used gastro-pancreatic digests for identifying the transported peptides. Our findings suggest that gastric digestion of major milk allergens preserves protein regions that might, upon pancreatic digestion, encounter immune system.

The importance of IgE binding to short, linear peptides derived from structured allergens such as Ara h 2 (peanut) or Pen a 1 (shrimp) in allergy management has been disputed [35]. Recently, in gastric-simulated digests of whole peanuts, 2S albumins were found to be the main contributors to IgE-reactivity against peanut SDRPs [19]. Therefore, pepsin-generated SDRPs may contain additional structural features to the solely IgE-binding epitope that facilitate partial refolding of the local structure and/or aggregation and adoption of the allergen-like conformation. Moreover, the aggregation tendency of digestion-derived peptides has been considered critical for the induction of allergic response in vivo [12,36]. Because caseins lack organized secondary structure upon encountering acidic environment in the stomach, the relevance of IgE binding to the linear epitopes (i.e., SDRPs) is high and should not be overlooked.

Here, we have shown that the peptides obtained by digestion of milk can form non-covalent aggregates comprising ≥4 SDRPs. Most of the identified SDRPs represented only part of the known IgE binding epitopes (Figure 4). However, the ability to inhibit IgE and provoke functional response in allergic individuals (Figure 5A,B) could clearly be attributed to the propensity of SDPRs to aggregate. The pronounced inhibition of IgE binding to caseins (Figure 5B) by SDRPs was in line with the fact that identified peptides derived mostly from caseins (more than 90%) (Figure 2A).

Although resistance to proteolysis is postulated as one of the main criteria for the risk assessment of the allergenicity of novel food proteins [8], it is becoming clear that even protein fragments obtained by digestion may still possess allergenic properties [12,23]. Whey protein hydrolysates with a molecular weight of 3–5 kDa can induce immune and allergic responses [21]. The minimal length of a peptide that can crosslink two IgE molecules anchored on the FcεRI on the surface of the mast cells was suggested to be 30 amino acids (~3 kDa) [37]. The majority of the peptides we have detected were below that size (approximately 1.2 kDa), yet the allergic response was induced in milk-allergic individuals (Figure 5B). Our results proved that milk SDRPs, which corresponded to a single IgE-binding epitope in size or to only a part of an IgE-binding epitope, preserved the ability to elicit allergic response due to their aggregation propensity (Figure 4). Moreover, our results suggest that even for the milk formulas based on hydrolysates containing peptides of a molecular weight less than 1.5 kDa, which are usually regarded as hypoallergenic [4,5,22,38], allergic reaction might be expected in subjects with CMA.

It has been suggested that the balance between milk-specific IgE and IgG4 plays a major role in tolerance induction [39]. IgE and IgG4 antibodies of children with transient CMA have been shown to recognize the same epitopes more often than antibodies from children with persistent CMA. Those results suggest that the overlap between IgE and IgG4 might be important in natural tolerance acquisition [39,40] and that the most relevant factor for tolerance development may be the capacity of IgG4 to bind to the same epitopes as IgE [41].

We could not confirm the role of SDRPs in milk-tolerant individuals, but IgG4 inhibition by SDRPs was found in three of the five examined SPT-positive individuals with CMA. Two sera that did not show IgG4 binding to SDRPs had a dominant IgE response to caseins (>70%) and four of the IgG4 tested patients also responded positively in SPT to SDRPs. A small group of CMA patients was tested but the absence of IgG4 recognition was demonstrated in half of the tests. Therefore, milk SDRPs could be part of the IgE-binding epitopes relevant in persistent CMA, as they induced allergic response by cross-linking the effector cells but lacked the ability to bind IgG4 from two out of four positive responders.

## 5. Conclusions

In summary, this is the first study to demonstrate that very short milk peptides comprise IgE-binding epitopes that can aggregate and re-form functional IgE binding epitopes. Most of the digestion-resistant peptides derived from caseins and were comprised of 7–14 amino acid sequences that correlated with those of major IgE-binding epitopes, but did not fully overlap with IgG4 epitopes. Major cow’s milk allergens ALA and BLG were mostly preserved as intact proteins. Short digestion-derived peptides from milk bound to IgE and elicited allergic reaction in sensitized subjects due to the aggregation.

## Supplementary Materials

The following are available online at https://www.mdpi.com/2304-8158/9/11/1576/s1. Supplementary Information. Table S1: IgE levels of patients used in the study determined by ImmunoCAP, Figure S1: Digestion of BLG at pH 1.2, 2.5 and 4.0, Figure S2: Digestion of ALA at pH 1.2, 2.5 and 4.0, Figure S3: MALDI spectra of peptides used in the study.
