# Supplementary material for: Digestomics of Cow’s Milk: Short Digestion-Resistant Peptides of Casein Form Functional Complexes by Aggregation
Source: Foods. 2020 Oct 30;9(11):1576. doi: 10.3390/foods9111576 (PMC7692894; doi:10.3390/foods9111576)
Supplement: Supplementary file 1 [file foods-09-01576-s001.zip › Figure S1-S3.pdf]

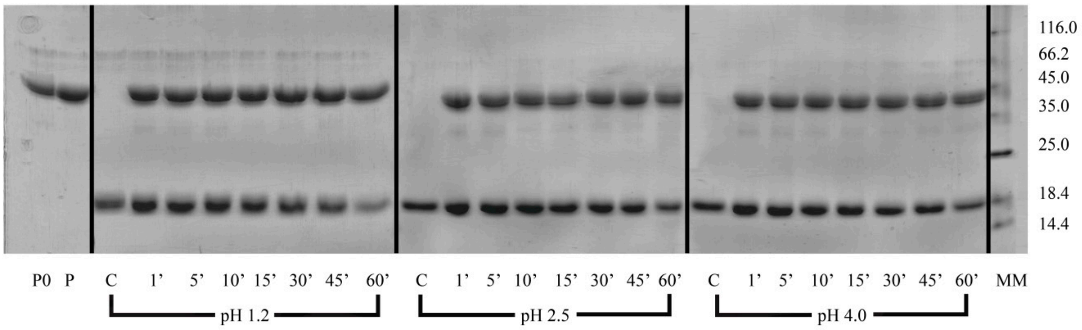

Figure S1: Digestion of BLG at pH 1.2, 2.5 and 4.0: P0—pepsin control, P—autoproteolysis of pepsin at pH=1.2 after 60 min, C—BLG only, 1′, 5′, 10′, 15′, 30′, 45′, 60′—aliquots taken at indicated timepoints, MM—molecular markers.

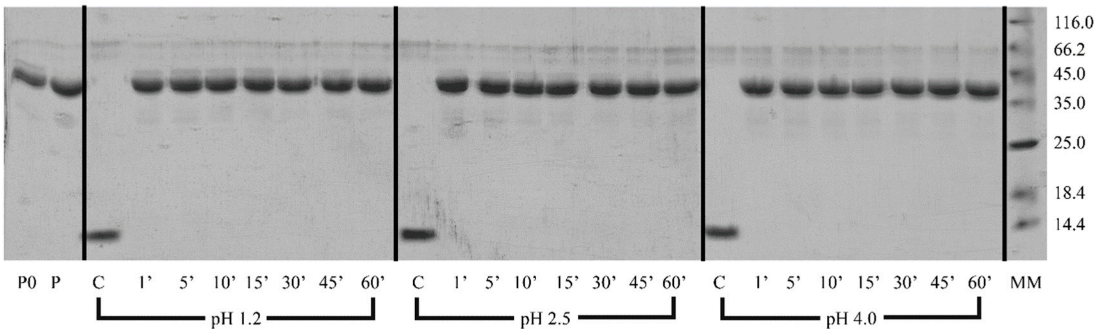

Figure S2: Digestion of ALA at pH 1.2, 2.5 and 4.0: P0—pepsin control, P—autoproteolysis of pepsin at pH=1.2 after 60 min, C—ALA only, 1′, 5′, 10′, 15′, 30′, 45′, 60′—aliquots taken at indicated timepoints, MM—molecular markers.

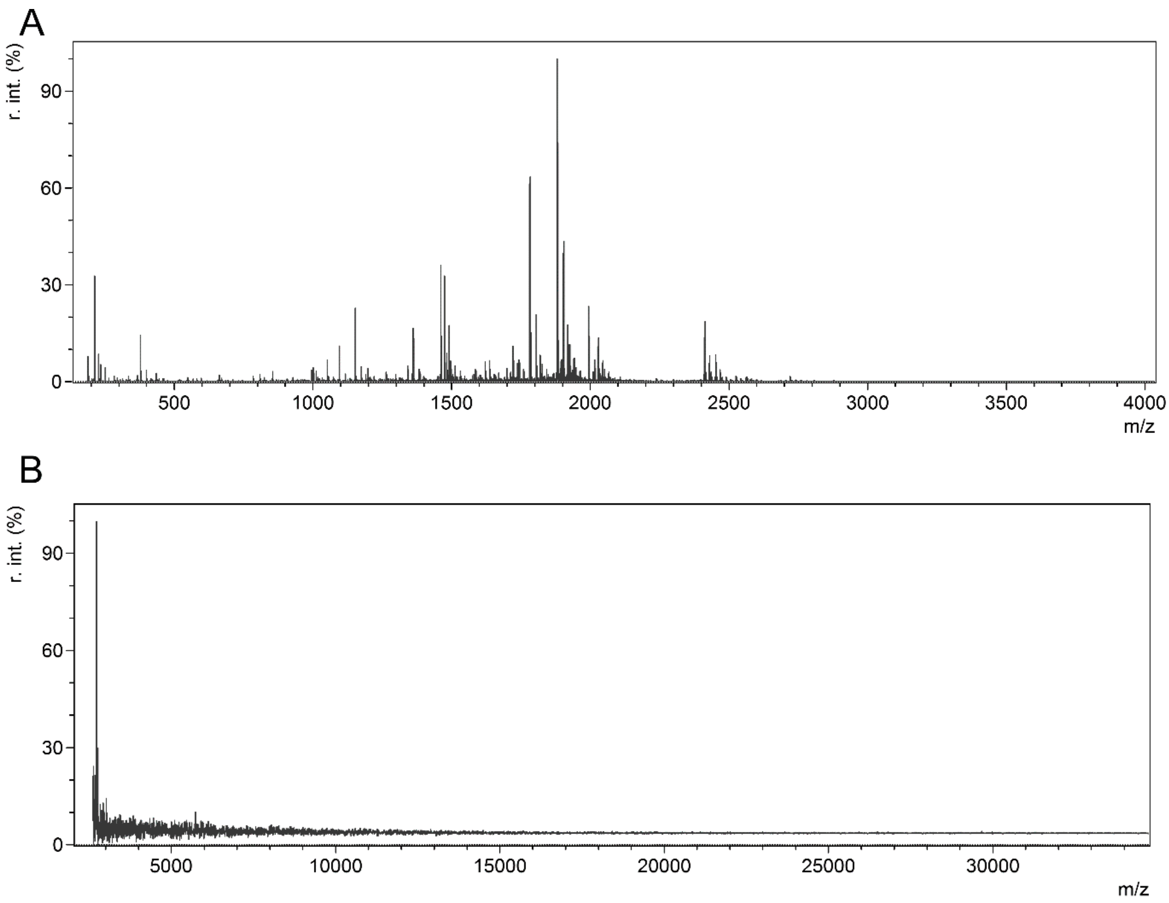

Figure S3: MALDI spectra of peptides used in the study. A—spectra obtained in positive ion reflector mode (peaks with m/z<1000 are mainly from matrix), B—spectra obtained in positive ion linear mode.
