# Supplementary material for: Digestomics of Cow’s Milk: Short Digestion-Resistant Peptides of Casein Form Functional Complexes by Aggregation
Source: Foods. 2020 Oct 30;9(11):1576. doi: 10.3390/foods9111576 (PMC7692894; doi:10.3390/foods9111576)
Supplement: Supplementary file 1 [file foods-09-01576-s001.zip › foods-901248-supplementary.pdf]

Supplementary Information

Methods

1.1 Detection of ALA and BLG by immunoblot

Stopped digests were mixed with reducing sample buffer (ratio 4:1) and boiled for 5 min at 95 °C. After resolving on 16% PAA gel along with molecular weight markers (Fermentas, Vilnius, Lithuania), samples were transferred to nitrocelullose membrane by semi-dry transfer system (Serva, Heidelberg, Germany). Membrane was blocked with 4% gelatine in TTBS (30 mM Tris pH 7.4 with 0.9% NaCI and 0.1% Tween 20) overnight and incubated with polyclonal rabbit anti-whey antibodies diluted 10 000 times in 1% gelatin in TTBS for 2 hours. Binding of anti-whey antibodies was detected by anti-rabbit antibody coupled to alkaline phosphatase (ABD Serotec, Oxford, United Kingdom) diluted in 1% gelatin in TTBS according to manufacturer's recommendation. For visualization solution containing 1.5 mg 5-bromo-4-chloro-3-indolyl phosphate (BCIP) and 3 mg nitro blue tetrazolium (NBT) in 10 mL of 100 mM Tris buffer containing 150 mM NaCI and 5 mM $MgCl_2$, pH 9.6 was used.

1.2 Mass spectrometry analysis

Chromatography was performed on a C18 nanocolumn (Thermo Scientific, Bremen, Germany; I.D. 75 μm, length 100 mm, particle size 2.2 μm) with a linear solvent gradient (5–70% B over 60 min, A: 0.1% formic acid; B: 0.1% formic acid in 2% acetonitrile). Spray voltage was adjusted to 1.8 kV, with a stainless steel emitter. Capillary temperature was set to 275 °C. Fourier transform mass spectrometry resolution was set to 30,000, with data-dependent acquisition of top five monoisotopic peaks, for m/z of 300–4,000. Dynamic acquisition with mass tolerance of 10 ppm was enabled. Data were analyzed by PEAKS Studio version 7.5 (Bioinformatics Solutions Inc, Waterloo, ON, Canada) against in-house FASTA database obtained by merging databases of milk proteins available from UniProt and cRAP (The common Repository of Adventitious Proteins) databases (both accessed on 10.07.2019.). Peptide tolerance and MS/MS tolerance were set to 10 ppm and 0.5 Da, respectively, with allowed variable modifications of

deamidation of Gln and Asn, oxidation of Met, His, Thr, and phosphorylation of Ser, Thr, and Tyr, with 5 as a maximal allowed number of variable PTMs per peptide. Pepsin at pH>2 was selected as cleavage protease with maximal missed cleavages of 100 and non-specific cleavage allowed at both ends. Identified peptides were aligned to main milk allergen sequences using web-based application MS Tools [1].

1.3 Size-exclusion chromatography

Aliquots of peptides were analyzed by size-exclusion chromatography (SEC). Briefly, lyophilized samples were reconstituted in the same volume of MiliQ water and 10 µL were applied to an analytical Superdex 75 3.2/30 column connected to the ÄKTA purifier 10 system (GE Healthcare, Chicago, IL, USA). Samples were eluted with phosphate buffered saline (137 mM NaCl, 2.7 mM KCl, 4.3 mM Na_2HPO_4, 1.47 mM KH_2PO_4, pH = 7.4) at 0.1 mL/min. The elution was monitored by measuring absorbances at 215 and 280 nm. The column was calibrated by ferritin (440 kDa), bovine serum albumin (66 kDa), ovalbumin (45 kDa), cytochrome C (14 kDa), aprotinin (6.5 kDa), and vitamin B12 (1.3 kDa)

1.4 IgG4-binding properties of peptides obtained by digestion

Ninety-six-well plate were coated with 100 µL/well of 2 µg/mL α-, β- or κ-casein in coating buffer (IgG4 Human Uncoated ELISA Kit with Plates (Cat. No # 88-50590-22 Invitrogen, Life Technologies Corporation, Carlsbad, USA) overnight at 4 °C. After double wash step, the wells were blocked with 250 µL blocking buffer over 2 hours at room temperature. Wells were washed twice and 100 µL of 20-fold diluted sera in assay buffer preincubated with PP or MP in 1:1 (vol:vol) ratio were added to the wells. Following 2h incubation at room temperature and four washes, wells were incubated with detection antibody according to the manufacturer's recommendations. After visualization with TMB as a substrate, A_{450} was measured. All measurements were done in duplicates.

1.5 Digestion of purified ALA and BLG at different pH

ALA and BLG were purified as previously described [2,3]. Digestion by pepsin was performed according to the previously published protocol for *in vitro* digestion of purified proteins [4]. Briefly, purified proteins were added at a final concentration of 0.25 mg/mL to 1.6 mL of simulated gastric fluid containing 2,300 U/mL pepsin (Sigma-Aldrich, Munich, Germany). For preparing simulated gastric fluid of pH=1.2, 0.1 M HCl with 2 g/L sodium chloride was used [4]. For pH=2.5 and pH=4.0, 0.1 M glycine and sodium-acetate buffers with corresponding pH values were used, respectively. Samples were incubated at 37 °C for 1, 5, 10, 15, 30, 45 and 60 min and aliquots of 32 μL were taken and the reaction was stopped by adding 10 μL of 2 M sodium bicarbonate, followed by adding 10 μL of 5 x reducing Laemmli buffer and boiling for 5 min at 95 °C. Controls had no pepsin added. Simultaneously, pepsin autoproteolysis was assayed at pH=1.2. After digestion, the samples (26 μL of each) were analyzed on 14 % PAA gels, using Tris-glycine buffer system. The results of BLG digestion are given in Figure S1, while ALA digestion profile is given in Figure S2.

1.6 MALDI-TOF MS

The MALDI-TOF MS experiments were performed on an UltrafleXtreme MALDI-TOF/TOF instrument (Bruker Daltonik, Leipzig, Germany) equipped with 1 kHz smartbeam II laser. Prior to the MS analysis, MP samples were desalted using C18 Zip-Tip pre-packed micro-columns (Millipore, Bedford, MA, USA) previously equilibrated with aqueous 0.1 % TFA (v/v) and eluted with 50 % acetonitrile (v/v) containing 0.1 % TFA (v/v). The mass spectra were acquired in the positive ion reflector mode (m/z = 0—4000) and in the positive ion linear mode (m/z = 2500—35000) by accumulating 2000 pulses with the accelerating voltage of 20 kV. Matrix for measurement in the reflector mode was α-cyano-4-hydroxycynnamic acid (in 97 % acetone, 0.1 % TFA), while for linear mode *trans*-3,5-dimethoxy-4-hydroxycinnamic acid (in 50 % acetonitrile, 0.1 % TFA) was used. The spectra were visualized by MSreView software (version 2.0).

Table S2 is provided separately as .xls file. It compares identified peptides from *in vitro* digestion and peptides derived from milk proteins which are detected in Caco-2 monolayer effluents [5]. For β-casein peptides identified from digestion with simulated and human oral and gastro-intestinal fluids are included [6].

References

1. Kavan, D.; Man, P. MSTools - Web based application for visualization and presentation of HXMS data. *Int J Mass Spectrom* **2011,** *302*, 53-58.
2. Al-Hanish, A.; Stanic-Vucinic, D.; Mihailovic, J.; Prodic, I.; Minic, S.; Stojadinovic, M.; Radibratovic, M.; Milcic, M.; Velickovic, T. C. Noncovalent interactions of bovine alpha-lactalbumin with green tea polyphenol, epigalocatechin-3-gallate. *Food Hydrocoll* **2016,** *61*, 241-250.
3. Stojadinovic, M.; Burazer, L.; Ercili-Cura, D.; Sancho, A.; Buchert, J.; Cirkovic Velickovic, T.; Stanic-Vucinic, D. One-step method for isolation and purification of native beta-lactoglobulin from bovine whey. *J Sci Food Agric* **2012,** *92*, 1432-1440.
4. Thomas, K.; Aalbers, M.; Bannon, G. A.; Bartels, M.; Dearman, R. J.; Esdaile, D. J.; Fu, T. J.; Glatt, C. M.; Hadfield, N.; Hatzos, C.; Hefle, S. L.; Heylings, J. R.; Goodman, R. E.; Henry, B.; Herouet, C.; Holsapple, M.; Ladics, G. S.; Landry, T. D.; MacIntosh, S. C.; Rice, E. A.; Privalle, L. S.; Steiner, H. Y.; Teshima, R.; Van Ree, R.; Woolhiser, M.; Zawodny, J. A multi-laboratory evaluation of a common in vitro pepsin digestion assay protocol used in assessing the safety of novel proteins. *Regul Toxicol Pharmacol* **2004,** *39*, 87-98.
5. Picariello, G.; Iacomino, G.; Mamone, G.; Ferranti, P.; Fierro, O.; Gianfrani, C.; Di Luccia, A.; Addeo, F. Transport across Caco-2 monolayers of peptides arising from in vitro digestion of bovine milk proteins. *Food Chem* **2013,** *139*, 203-212.
6. Benede, S.; Lopez-Exposito, I.; Gimenez, G.; Grishina, G.; Bardina, L.; Sampson, H. A.; Molina, E.; Lopez-Fandino, R. In vitro digestibility of bovine beta-casein with simulated and human oral and gastrointestinal fluids. Identification and IgE-reactivity of the resultant peptides. *Food Chem* **2014,** *143*, 514-521.
