# Supplementary material for: Digestomics of Cow’s Milk: Short Digestion-Resistant Peptides of Casein Form Functional Complexes by Aggregation
Source: Foods. 2020 Oct 30;9(11):1576. doi: 10.3390/foods9111576 (PMC7692894; doi:10.3390/foods9111576)
Supplement: Supplementary file 1 [file foods-09-01576-s001.zip › Table S1.pdf]

Table S1. IgE levels of patients used in the study determined by ImmunoCAP

Patient ′s ID	Patient′s nationality	milk (kAU/L)	ALA (kAU/L)	BLG (kAU/L)	casein (kAU/L)	Serum dilution for IgE inhibition (fold)
1	Sweden	17	-	-	-	2
2	Sweden	27	-	-	-	2
3	Sweden	97	45	25.2	65.7	9
4	Sweden	22	12.4	4.8	14.8	2
5	Sweden	22	20	3.6	4.2	2
6	Sweden	88	13.6	11.2	74.4	8
7	Sweden	43	33.6	15.6	18.4	4
8	Sweden	56	3.5	9.5	175	5
9	Sweden	25	26	3.8	7	2
10	Sweden	26	10.2	6	4.6	2
11	Sweden	46	5.6	1.8	10.8	4
12	Serbia	192	33.6	88	112	8
13	Serbia	0.37	0.17	<0.1	0.25	
14	Serbia	7.5	1.0	1.7	7.2	
15	Serbia	0.19	<0.1	<0.1	0.13	
16	Serbia	73	21	6.4	57	
17	Sweden	n.d.*	n.d.	n.d.	<0.1	
18	Sweden	n.d.	n.d.	n.d.	<0.1	

19	Sweden	n.d.	n.d.	n.d.	<0.1
20	Sweden	n.d.	n.d.	n.d.	<0.1
21	Sweden	n.d.	n.d.	n.d.	<0.1
22	Serbia	N/A	N/A	N/A	N/A
23	Serbia	N/A	N/A	N/A	N/A

*n.d.—not detected by ISAC ImmunoCAP (<0.3 ISU-E); N/A—no data available
